# Microscopic cues shape neuronal morphology and microcircuits

**DOI:** 10.1186/1471-2202-15-S1-P180

**Published:** 2014-07-21

**Authors:** Benjamin Torben-Nielsen, Erik De Schutter

**Affiliations:** 1 Computational Neuroscience Unit, Okinawa Institute of Science and Technology Graduate University, 1919-1 Tancha, Onna-son, Kunigami-gun, Okinawa, Japan 904-0495

## 

Neurons grow in a dense brain matrix and interactions with this matrix shape their adult morphology and the resultant microcircuit. From experimental work it is known that many factors regulate these interactions and influence neuronal morphology at a microscopic scale [1]. However, it remains largely unknown how a combination of these factors propagate to overall morphology and microcircuits.

In this work we address how microscopic interactions shape neuronal morphology and lead to the formation of microcircuits. By using a computational model we can single out individual growth factors and calculate their propagation from microscopic interactions to the macroscopic realization of the neuronal circuit.

We propose a software framework to develop large numbers of virtual neuronal morphologies that interact with each other and the brain matrix during their simulated growth. Development is simulated at a phenomenological level by repetitively extending “fronts”, the computational equivalents of growth cones [2]. Fronts have a bi-directional interaction with the surrounding matrix. On the one hand, fronts can query the matrix and extended (or not) based on local information about attractors and repellants. On the other hand, fronts can secrete “environmental cues” that update the matrix and can be used by other fronts (or by itself) as guidance signal. At the time of simulated growth, putative synapse locations at structural appositions can be recorded to construct microcircuits.

During simulated growth, the matrix is decomposed into sub-volumes that are distributed over multiple computer processors and to which fronts are assigned. This spatial decomposition exploits the local nature of growth cone signaling to ensure scalability with increasing numbers of computer cores.

We are validating the framework and are working on preliminary simulations in which we address planarity and tiling in populations of Purkinje neurons.
